# Supplementary material for: Activating silicon for high hydrogen conversion and sustainable anode recovery
Source: Nat Commun. 2025 Aug 20;16:7772. doi: 10.1038/s41467-025-63086-x (PMC12368011; doi:10.1038/s41467-025-63086-x)
Supplement: Supplementary file 1 — Supplementary Information [file 41467_2025_63086_MOESM1_ESM.pdf]

Supplementary Materials for

Activating Silicon for High Hydrogen Conversion and Sustainable Anode Recovery

Mili Liu[1#], Yunqi Jia[1#], Jiangwen Liu[1], Kang Chen[1], Hao Zhong[1], Lin Jiang[2]*, Hui Liu[1,3]*, Liuzhang Ouyang[1,4*], Min Zhu[1*]

[1] School of Materials Science and Engineering, Guangdong Provincial Key Laboratory of Advanced Energy Storage Materials, South China University of Technology; Guangzhou, 510641, PR China.

[2] School of Microelectronics, Shanghai University; Shanghai, 201800, PR China.

[3] School of Chemistry and Material Science, Hunan Agricultural University; Changsha, 410128, PR China.

[4] Guangdong Engineering Technology Research Center of Advanced Energy Storage Materials; Guangzhou, 510641, PR China.

[#] These authors contributed equally to this work: Mili Liu and Yunqi Jia.

*Corresponding author.

Email: linjiang@shu.edu.cn, liu.hui@hunau.edu.cn, meouyang@scut.edu.cn, memzhu@scut.edu.cn

The file includes:

Supplementary Figs. 1 to 34

Supplementary Tables 1 to 6

Supplementary References 1-31

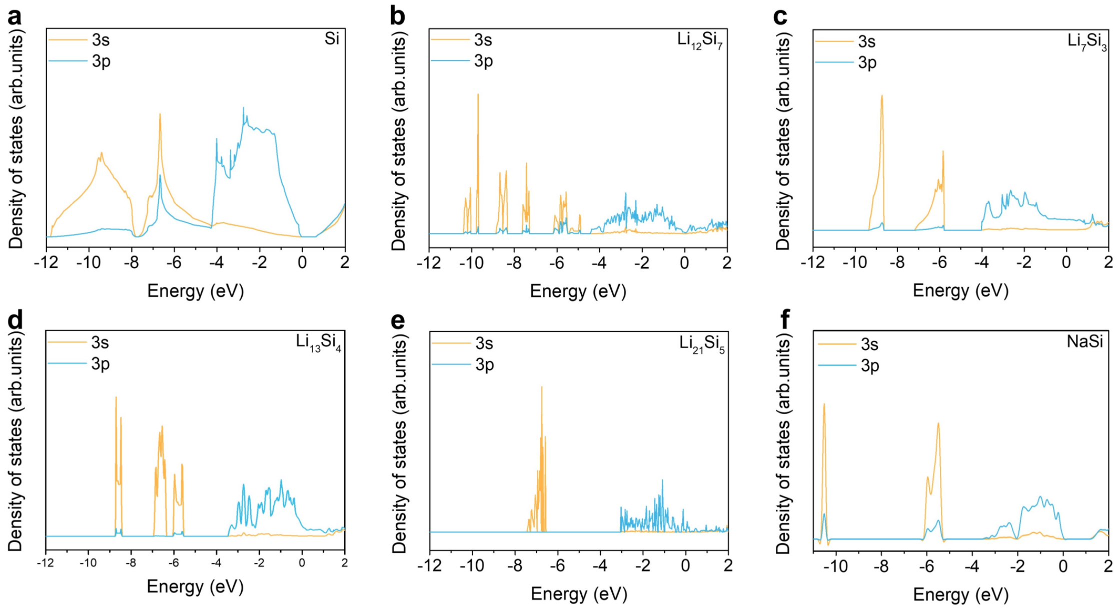

Supplementary Fig. 1 Density of states (DOS) projected on Si atoms in pure Si, four Zintl Li-Si alloys and Zintl NaSi alloy.

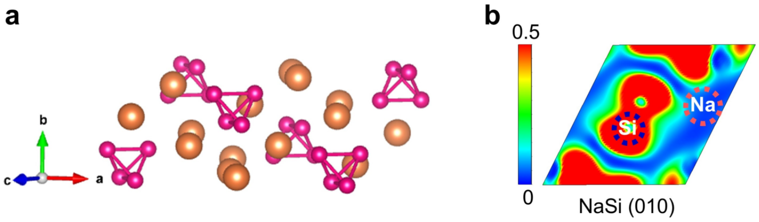

Supplementary Fig. 2 (a) Crystal structure of Zintl NaSi alloy and (b) the corresponding ELF image for the (010) lattice plane.

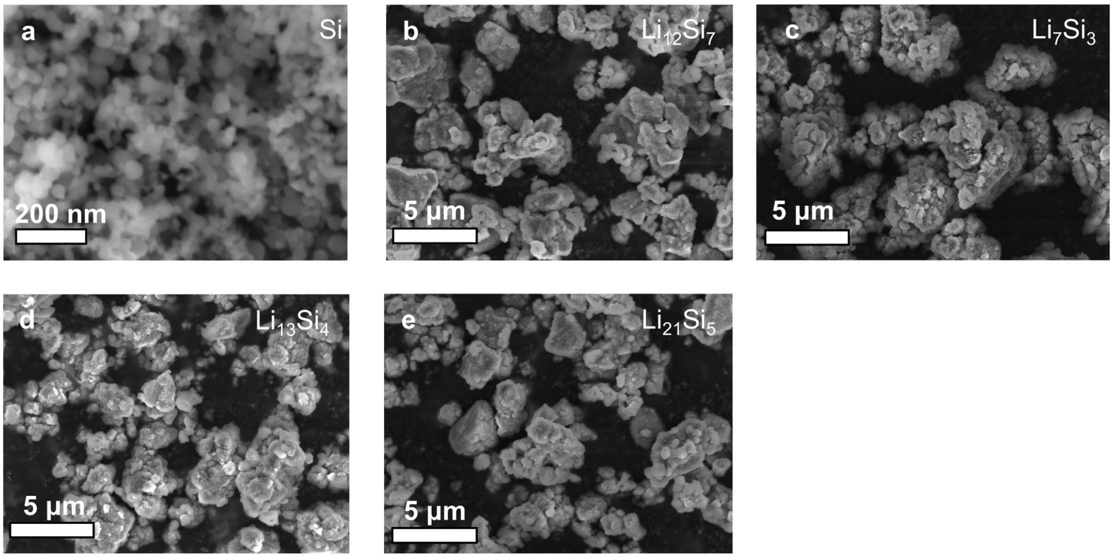

Supplementary Fig. 3 SEM images of Nano Si and four as-prepared Li-Si alloys.

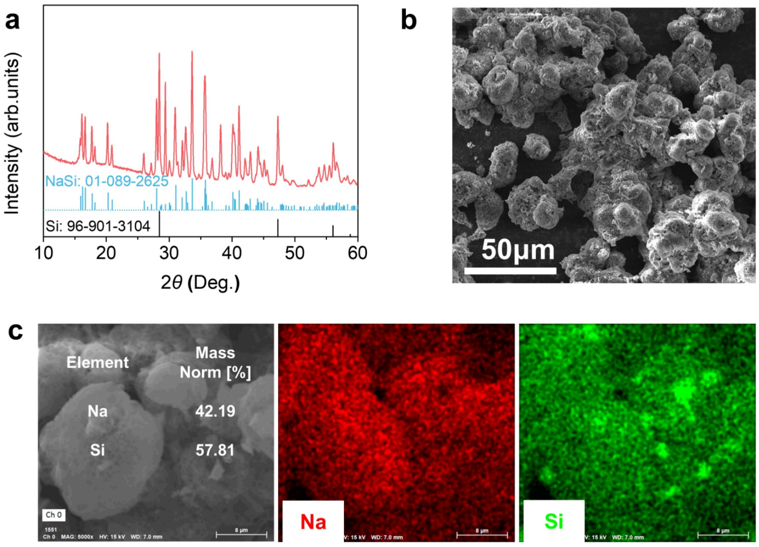

Supplementary Fig. 4 (a) XRD pattern, (b) SEM image and (c) EDS mapping images of as-prepared NaSi alloy.

Supplementary Note 1:

The as-prepared NaSi alloy shows a subglobular microstructure with a homogenous distribution of Na and Si, and the mass ratio of Na/Si was approximately 42:58.

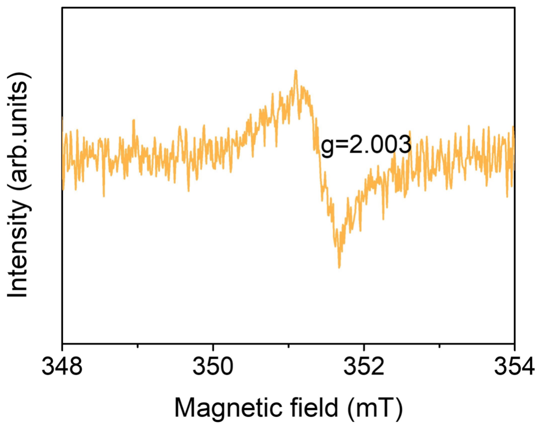

Supplementary Fig. 5 ESR spectra of as-prepared NaSi alloy.

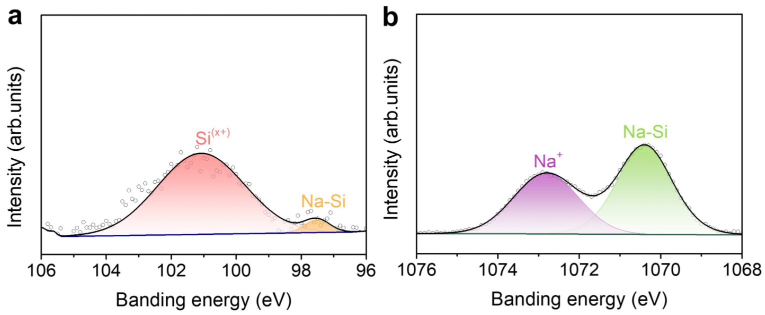

Supplementary Fig. 6 XPS spectra of (a) Si 2*p* and (b) Na 1*s* for as-prepared NaSi alloy.

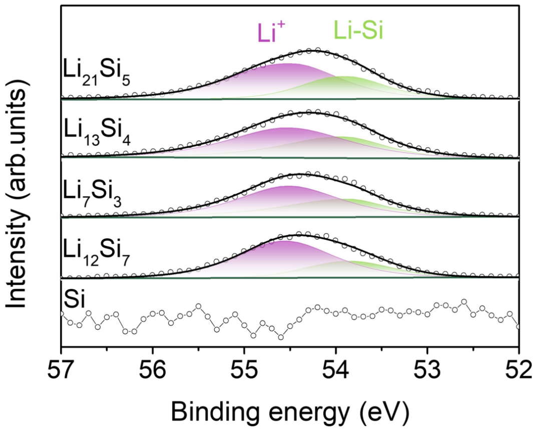

Supplementary Fig. 7 XPS spectra of Li 1*s* for nano Si and as-prepared Li-Si alloys.

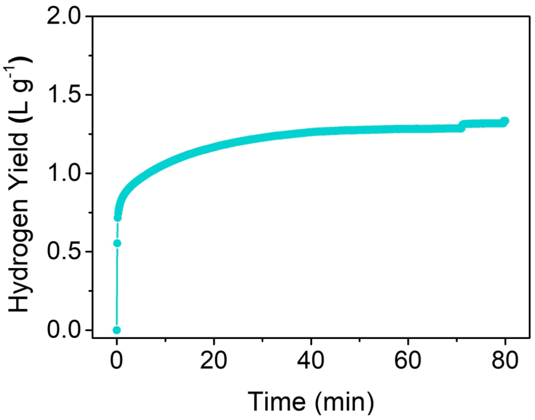

Supplementary Fig. 8 Hydrogen evolution curve of $Li_{12}Si_7$ alloy in pure water within 80 min at 25 °C.

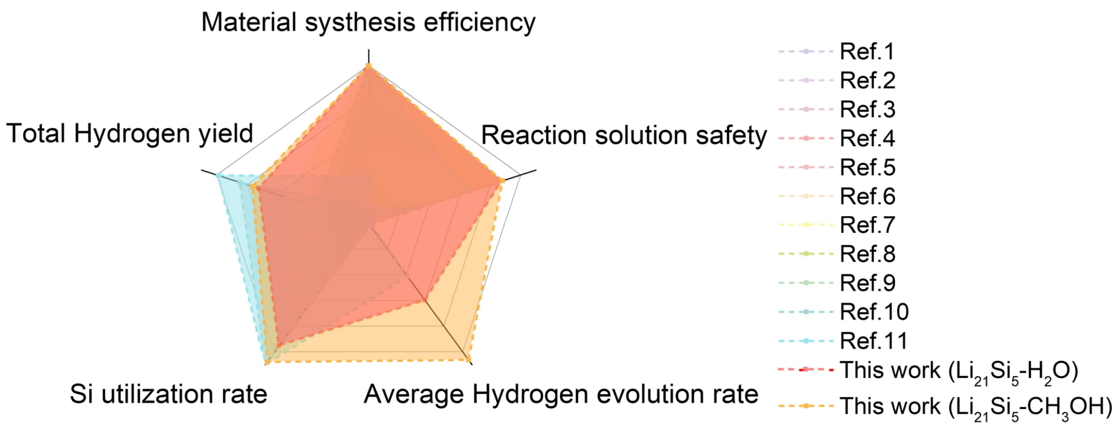

Supplementary Fig. 9 Comprehensive comparison of Zintl $Li_{21}Si_5$ alloy with other reported Si-based works.

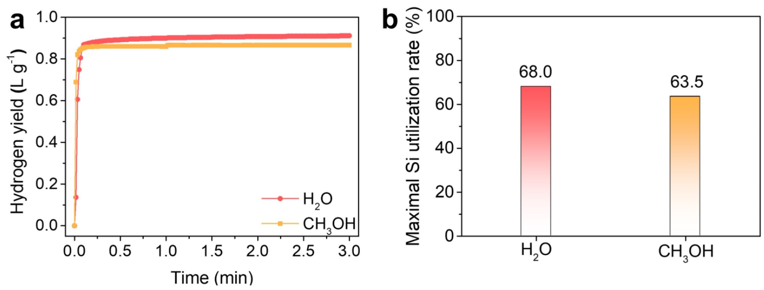

Supplementary Fig. 10 (a) Hydrogen evolution curves and (b) related Si utilization rates of as-prepared NaSi alloy in pure water and methanol at 25°C, respectively.

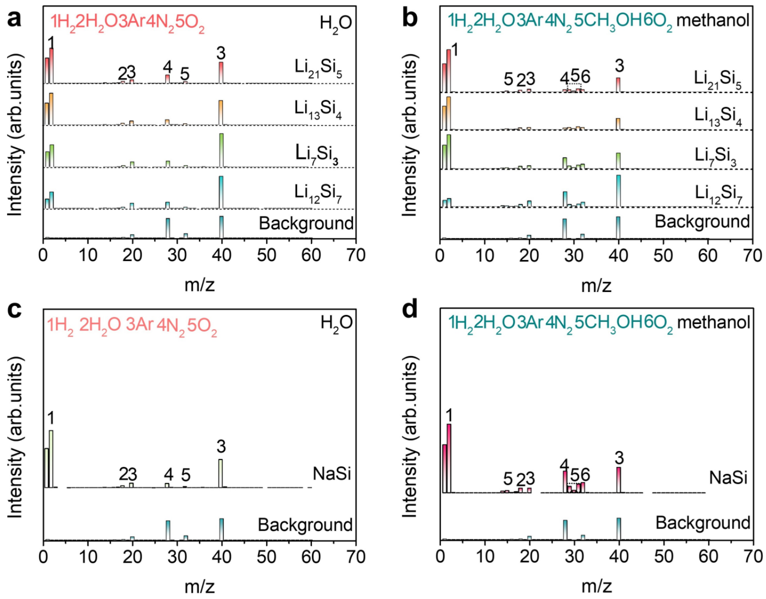

Supplementary Fig. 11 MS analysis of the gas produced by the Zintl Li-Si alloys in (a) pure water and (b) methanol, and by the as-prepared NaSi alloy in (c) pure water and (d) methanol.

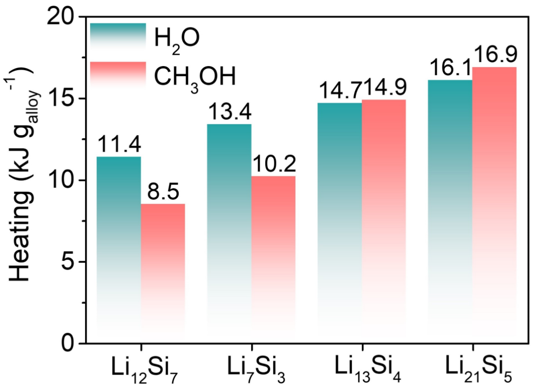

Supplementary Fig.12 Theoretical heating values released from different Zintl Li-Si alloys in pure water and methanol.

Supplementary Note 2:

Based on the hydrogen evolution performance of various Zintl Li-Si alloys in the pure water and methanol at 25°C (Fig. 2a-d), the corresponding reaction equations were summarized in the Supplementary Table 6 and the theoretical exothermicity of these reactions were calculated. As shown in Supplementary Fig. 12, 1g alloys of $Li_{21}Si_5$, $Li_{13}Si_4$, Li_7Si_3 and $Li_{12}Si_7$ in pure water and methanol can produce 16.1 kJ/ 16.9 kJ, 14.7 kJ/ 14.9 kJ, 13.4 kJ/ 10.2 kJ, 11.4 kJ/ 8.5 kJ of heat, respectively, highlighting the vigorously exothermic characteristic of these hydrogen production systems.

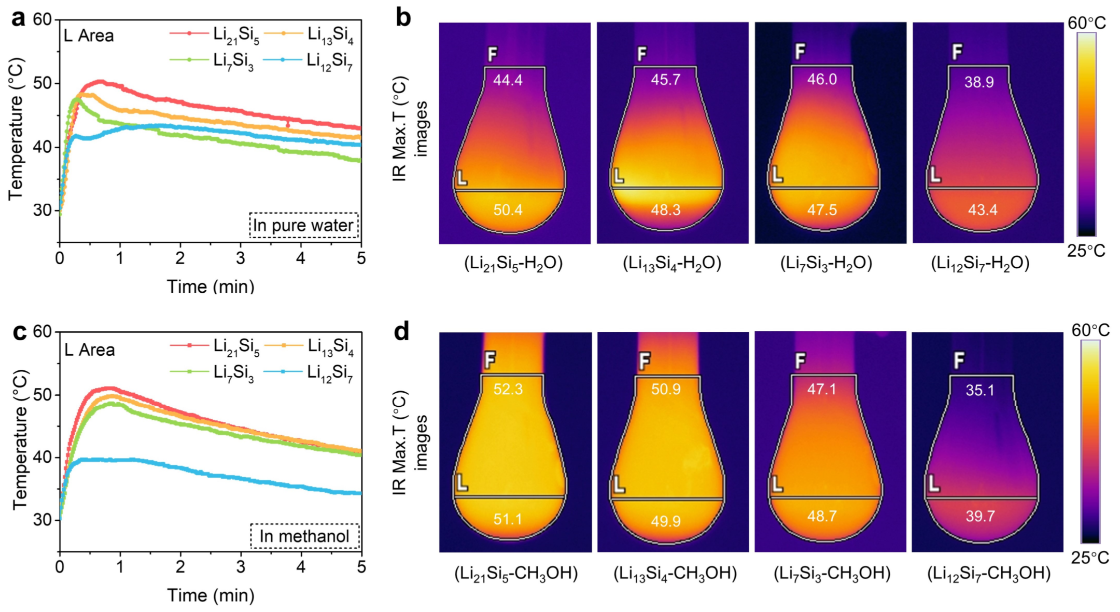

Supplementary Fig. 13 Temperature change curves of liquid (L) reaction area for various alloys and the IR images of the liquid areas and flasks with recorded maximum temperature: (a, b) hydrolysis systems and (c, d) methanolysis systems.

Supplementary Note 3:

We employed an infrared thermal camera to monitor practical temperature changes in both liquid reaction area (L area) and flask area (F area) of different hydrolysis and methanolysis reactions over a 5-minute timeframe, under the conditions of 10 ml solvent and 0.1 g alloy in a 20 ml flask. As shown in Supplementary Fig. 13a, the $Li_{21}Si_5$, $Li_{13}Si_4$, Li_7Si_3 and $Li_{12}Si_7$ alloys in pure water achieve peak L area temperatures of 50.4°C, 48.3°C, 47.5°C and 43.4°C, respectively, while corresponding flask temperatures don't exceed 46°C (Supplementary Fig. 13b). Similarly, methanol-mediated reactions also produce substantial heat release (Supplementary Fig. 13c, d). Notably, the $Li_{21}Si_5$-CH_3OH system registers the most pronounced thermal output, while the maximum temperatures of L area and F area are 51.1°C and 52.3°C, respectively. Moreover, the maximum L area temperatures and relevant flask temperatures of $Li_{13}Si_4$, Li_7Si_3 and $Li_{12}Si_7$ alloys in methanol are 49.9°C/50.9°C, 48.7°C/47.1°C and 39.7°C/35.1°C, respectively.

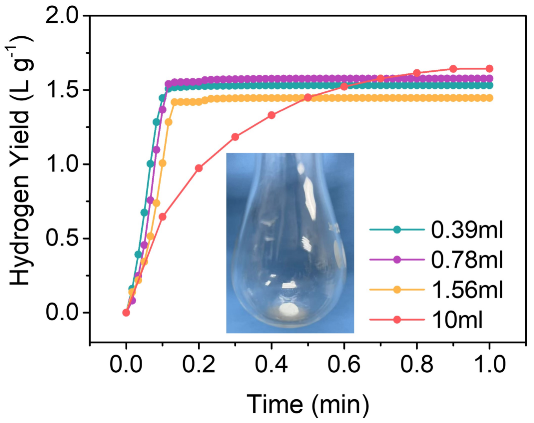

Supplementary Fig. 14 The hydrogen production curves of $Li_{21}Si_5$ alloy with various amount of pure water at 25°C, the inset image is the product of reaction system with 1.56 mL pure water.

Supplementary Note 4:

According to the theoretical reaction equation of $Li_{21}Si_5$ + 31H_2O → 21LiOH + 5SiO_2 + 20.5H_2, various stoichiometric amounts of pure water ($H_2O/Li_{21}Si_5$ = 62~248 mol/mol, equivalent to 0.39~1.56 mL H_2O/0.1 g $Li_{21}Si_5$) were added to react with $Li_{21}Si_5$ alloy at 25°C. As revealed in Supplementary Fig.14, under the water-restricted conditions, the $Li_{21}Si_5$ alloy demonstrates steep hydrogen generation kinetics during the initial 10-second process and delivers 1.532 L g^{-1}, 1.578 L $g^{-1}$and 1.446 L g^{-1} H_2 within 1 min in the 0.39 mL, 0.78 mL and 1.56 mL water, respectively. These hydrogen evolution behaviors show significant deviation from the performance observed in the much excess water condition (~10 ml water), particularly regarding reaction dynamics and ultimate hydrogen yield. The critical difference stems from water's bifunctional nature as both reactant and thermal buffer, while its limited quantity dramatically affects heat dissipation capacity during the exothermic hydrolysis process. The intensified thermal accumulation not only initiates a self-accelerating effect on the initial hydrogen release, but also produces uncontrolled local temperature escalation that induces partial melting of the alloy matrix and facilitates rapid precipitation of passivation products, further blocking water penetration and terminating the hydrolysis reaction prematurely. Especially, even the initial reactivity is slightly reduced when the water volume increases to 1.56 mL, the thermal runaway and passivation layer

formation still fail to be completely suppressed (as seen in the inset image), conversely resulting in a lower hydrogen yield. Comparatively, the water-excessive system (10 mL) demonstrates superior thermal regulation and maintains moderate temperature spikes. This thermal stabilization enables a higher reaction efficiency, yielding 1.643 L g^{-1} H_2 through sustained hydrolysis within 60 seconds.

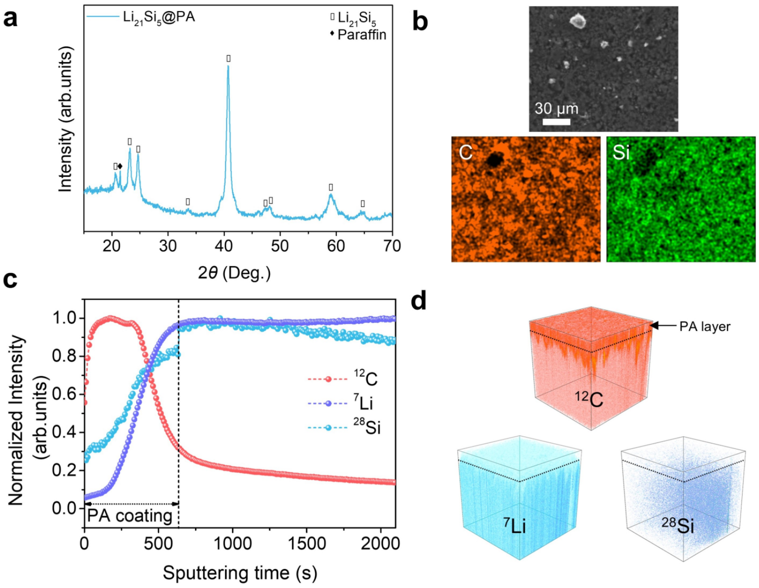

Supplementary Fig. 15 (a) XRD pattern of $Li_{21}Si_5$@PA composite. (b) EDS mapping of $Li_{21}Si_5$@PA composite. (c) The vertical distribution profiles of ^{12}C, ^{7}Li, and ^{28}Si signals and (d) corresponding three-dimensional mapping images of $Li_{21}Si_5$@PA composite.

Supplementary Note 5:

As shown in Supplementary Fig. 15a, the XRD pattern shows the peaks corresponding to $Li_{21}Si_5$ and PA, indicating decent compatibility between the active $Li_{21}Si_5$ alloy and PA coating. EDS mapping and TOF-SIMS results reveal the uniformity and distribution of PA coating layer. As shown in Supplementary Fig. 15b, surface EDS mapping demonstrates an even distribution of C and Si elements for the $Li_{21}Si_5$@PA. Moreover, TOF-SIMS depth profiling and three-dimensional images suggest that the dense PA coating layer is dominantly located on the outer surface of alloy matrix (Supplementary Figs. 15c, d). As a result, the well-defined coating structure would promote $Li_{21}Si_5$@PA composite to achieve splendid air stability.

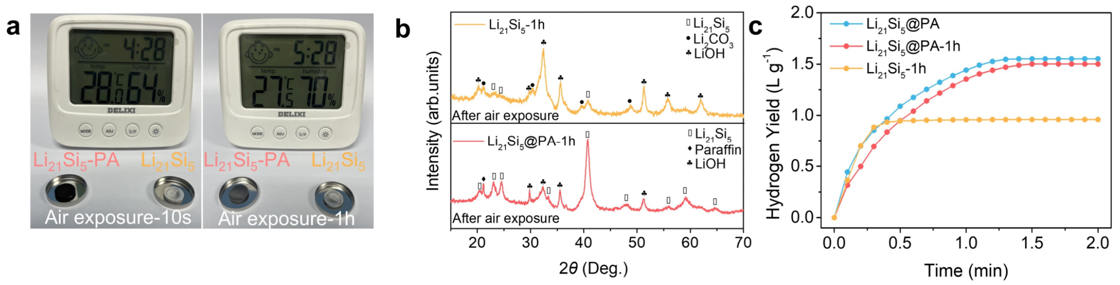

Supplementary Fig.16 (a) Digital photos of $Li_{21}Si_5$ alloy and $Li_{21}Si_5$@PA composite after air exposure within 10 s and 1 h; (b) XRD patterns of $Li_{21}Si_5$ alloy and $Li_{21}Si_5$@PA composite after air exposure within 1 h; (c) Hydrogen evolution curves of pristine $Li_{21}Si_5$@PA composite, $Li_{21}Si_5$ alloy and $Li_{21}Si_5$@PA composite after air exposure within 1 h.

Supplementary Note 6:

The air exposure tests of $Li_{21}Si_5$ alloy and $Li_{21}Si_5$@PA composite were conducted under ambient atmosphere (64-70% humidity, ~28°C). As shown in Supplementary Fig. 16a, severe structural degradation of $Li_{21}Si_5$ alloy is evident just after 10 s of air exposure. When the exposure time is prolonged to 1 h, the active $Li_{21}Si_5$ phase structure is almost poisoned, accompanied by the significant formation of LiOH and Li_2CO_3 (Supplementary Fig. 16b). In contrast, the $Li_{21}Si_5$@PA shows a much more stable structure, exhibiting only slight surface failure after the same 1-hour exposure period (Supplementary Fig. 16a, b). Therefore, while the initial hydrogen production kinetics of $Li_{21}Si_5$@PA is slightly lower than the $Li_{21}Si_5$ alloy (1.552 L g^{-1} H_2 in 2 min vs 1.643 L g^{-1} H_2 in 1 min), it achieves a significant enhancement of hydrogen yield retention from 58.4% to 96.6% (Supplementary Fig. 16c).

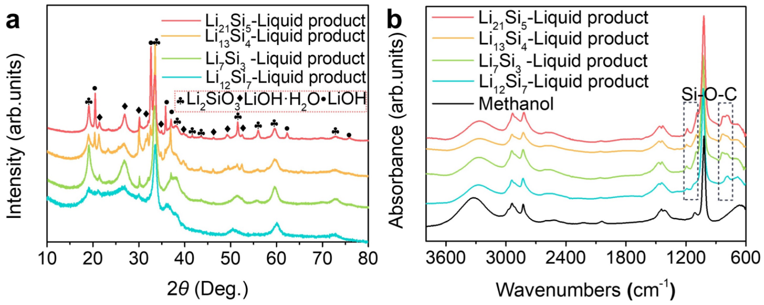

Supplementary Fig. 17 (a) XRD patterns of the dried hydrolysis liquid products for Zintl Li-Si alloys. (b) FTIR analysis of methanolysis liquid products for Zintl Li-Si alloys.

Supplementary Note 7:

The liquid products from both Zintl Li-Si hydrogen evolution systems after separating the unreacted Si were analyzed. It is shown that the hydrolysis liquid products contain LiOH and Li_2SiO_3, with the latter attributed to the reaction between LiOH and SiO_2 sol, as evidenced in Supplementary Fig. 17a. Meanwhile, the FTIR spectra of methanolysis liquid products in Supplementary Fig. 17b reveal characteristic IR absorption peaks of Si-OCH_3 structure at 1092, 1192, 831, and 789 cm^{-1} [1-3], which is further verified to be tetramethoxysilane (TMOS) by later gas chromatography-mass spectrometry (GC-MS) (Supplementary Fig. 18). Upon reacting with methanol, the lithium (Li) present in Li-Si alloys transforms into $LiOCH_3$.

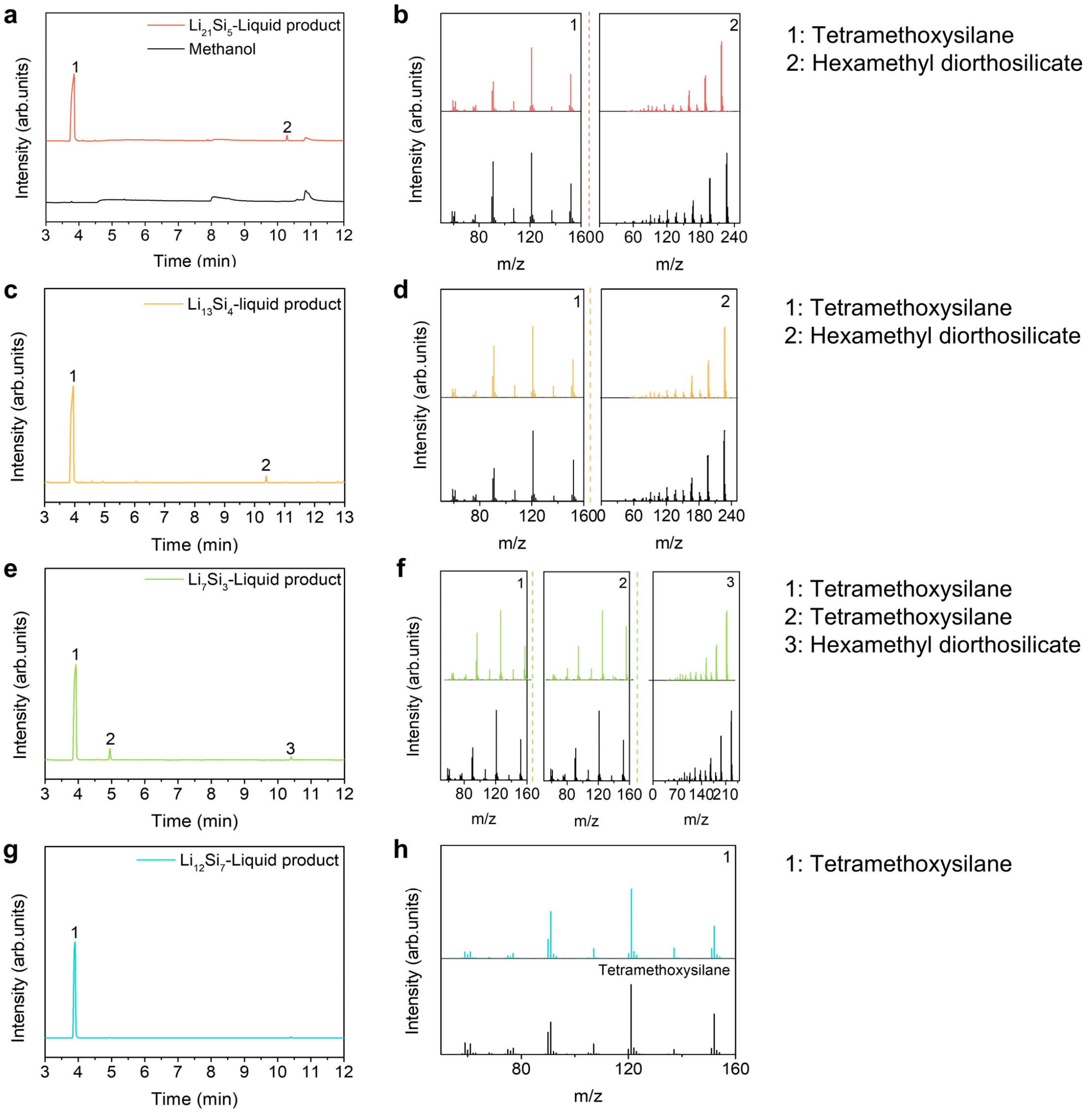

Supplementary Fig. 18 Chromatograms (left) and mass spectra (right) analysis of methanolysis liquid product for (a, b) $Li_{21}Si_5$, (c, d) $Li_{13}Si_4$, (e, f) Li_7Si_3 and (g, h) $Li_{12}Si_7$ alloys. The trace of hexamethyl diorthosilicate is attributed to the sensitive side reaction between TOMS and the water of air during the measurement process.

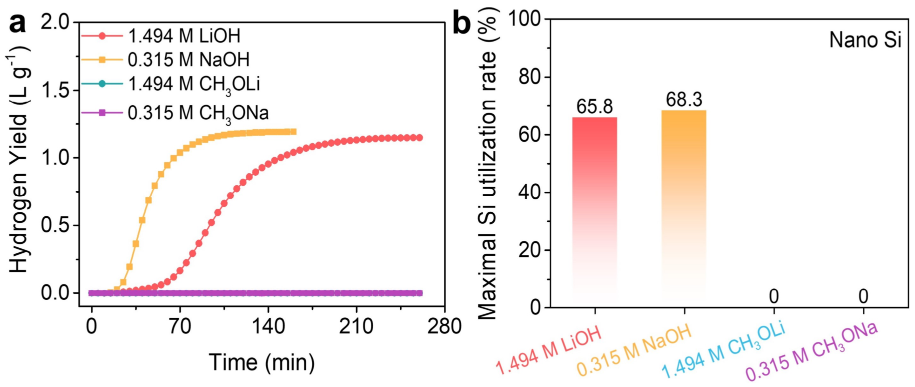

Supplementary Fig. 19 (a) Hydrogen evolution curves of Si nanoparticles in 1.494 M LiOH solution, 0.315 M NaOH solution, 1.494 M CH_3OLi solution and 0.315 M CH_3ONa solution and (c) the corresponding maximal Si utilization rates.

Supplementary Note 8:

Employing the synthesized Zintl $Li_{21}Si_5$ and NaSi alloys as references based on the principle of alkali metal atomic equivalence, we have tested the hydrogen generation performance of Si nanoparticles in both aqueous (1.494 M LiOH/0.315 M NaOH) and methanolic (1.494 M CH_3OLi/0.315 M CH_3ONa) alkaline media. The hydrogen production tests were carried out with 0.1 g Si nanoparticles and 10 ml reaction solution, while the reaction temperature was 25°C. As shown in Supplementary Fig. 19a, Si nanoparticles exhibit the induction durations of 20 min and 15 min in the LiOH and NaOH solutions, respectively, which is attributed to the dissolution process of surface oxide layer. Afterwards, the Si nanoparticles experience extremely slow hydrogen generation kinetics. It delivers 1.149 L g^{-1} H_2 and 1.192 L g^{-1} H_2 within the ultralong reaction durations of 235 minutes and 145 minutes, respectively, while the corresponding maximal Si utilization rates are 65.8% and 68.3% (Supplementary Fig. S19b). Meanwhile, the CH_3OLi and CH_3ONa solutions show no promoting effect on the hydrogen generation of Si. The huge difference of hydrogen evolution kinetics between Si-alkalis solutions systems and Zintl $Li_{21}Si_5$/NaSi alloys-H_2O/CH_3OH systems suggests that the ultra-fast and ultra-high Si-H_2 conversions of Zintl phases are dominantly attributed to the unpaired electron structure rather than the simple catalyzing of symbiotic alkali metals' products.

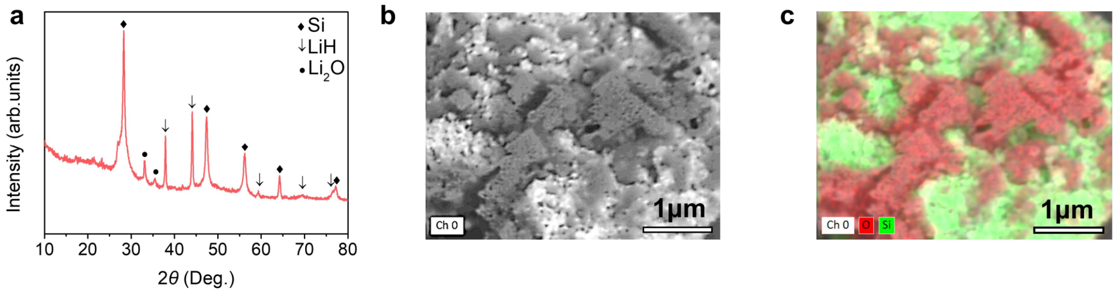

Supplementary Fig. 20 (a) XRD pattern, (b) BSE image and (c) element mapping image of H-$Li_{21}Si_5$.

Supplementary Note 9:

The hydriding of $Li_{22}Si_5$ alloy was conducted at 320°C under the hydrogen pressure of 5MPa within 25 h, resulting to the unoxidized Si dispersed in LiH matrix ($Li_{21}Si_5$ + 21/2H_2 → 21LiH + 5Si).

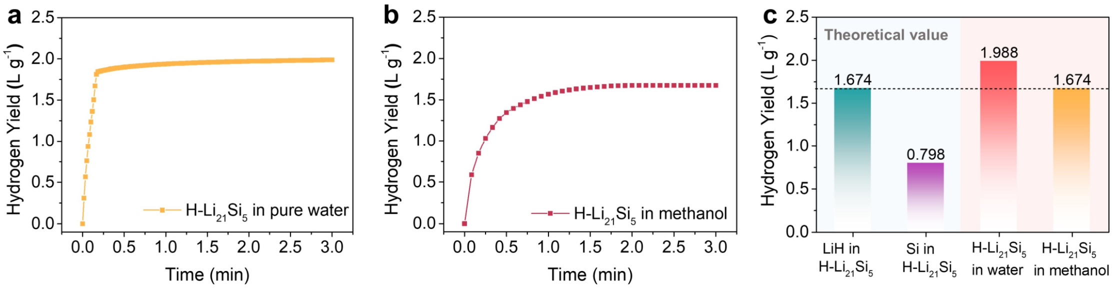

Supplementary Fig. 21 Hydrogen evolution curves of H-$Li_{21}Si_5$ in (a) pure water and (b) methanol. (c) Theoretical hydrogen contribution of LiH/Si in H-$Li_{21}Si_5$ and the hydrogen yields of H-$Li_{21}Si_5$ in pure water/ methanol.

Supplementary Note 10:

Hydrogen generation results indicate that the H-$Li_{22}Si_5$ liberates 1.988 L g^{-1} H_2 in water and 1.674 L g^{-1} H_2 in methanol, respectively (Supplementary Figs. 21a, b). The theoretical hydrogen contributions of LiH and Si in the H-$Li_{21}Si_5$ are 1.674 L g^{-1} and 0.798 L g^{-1}, respectively. Correspondingly, the Si utilization rates of H-$Li_{21}Si_5$ in water and methanol are 39.3% and 0% (Supplementary Fig. 21c), respectively, markedly lower than those of Zintl $Li_{21}Si_5$ alloy.

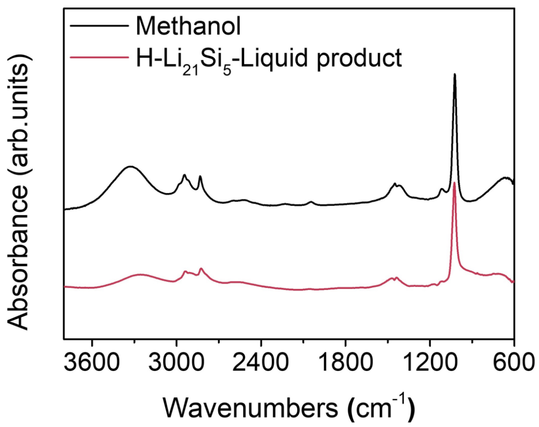

Supplementary Fig. 22 FTIR spectra of the methanol and the liquid product of the H-$Li_{21}Si_5$-methanol system.

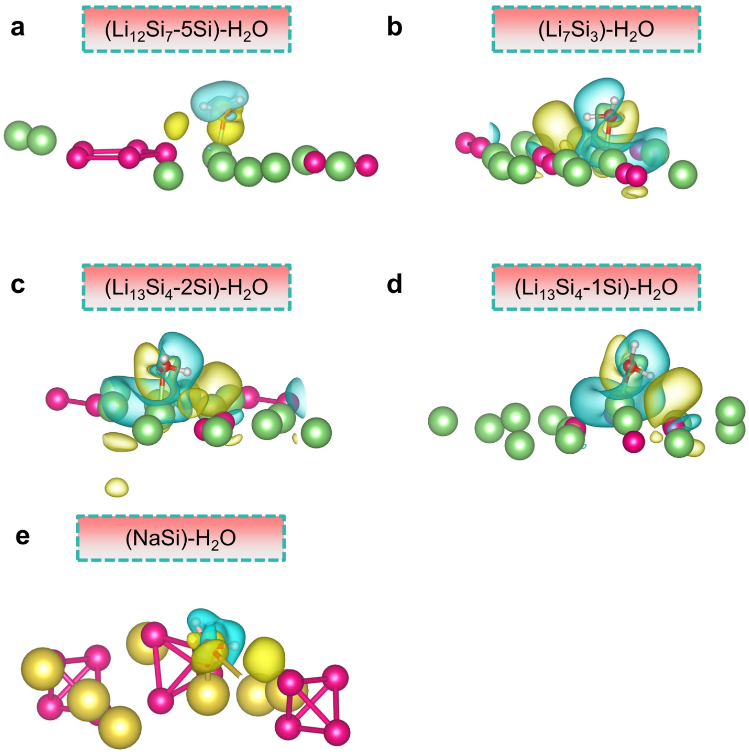

Supplementary Fig. 23 Difference density of electron distribution of configurations for the H_2O activation step. (a) $Li_{12}Si_7$-5Si, (b) Li_7Si_3, (c) $Li_{13}Si_4$-2Si, (d) $Li_{13}Si_4$-1Si, (e) NaSi.

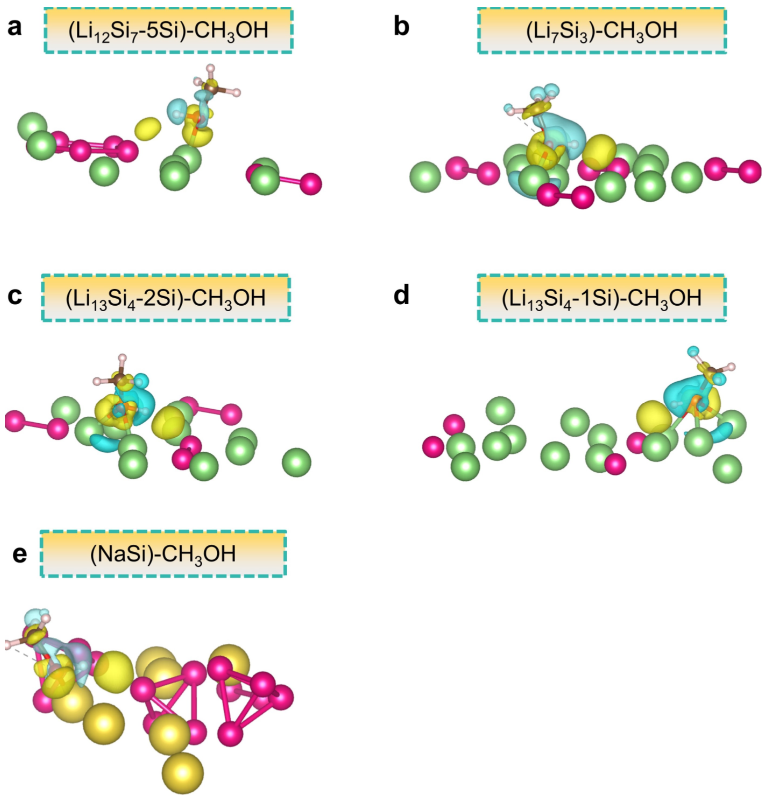

Supplementary Fig. 24 Difference density of electron distribution of configurations for the CH_3OH activation step. (a) $Li_{12}Si_7$-5Si, (b) Li_7Si_3, (c) $Li_{13}Si_4$-2Si and (d) $Li_{13}Si_4$-1Si, (e) NaSi.

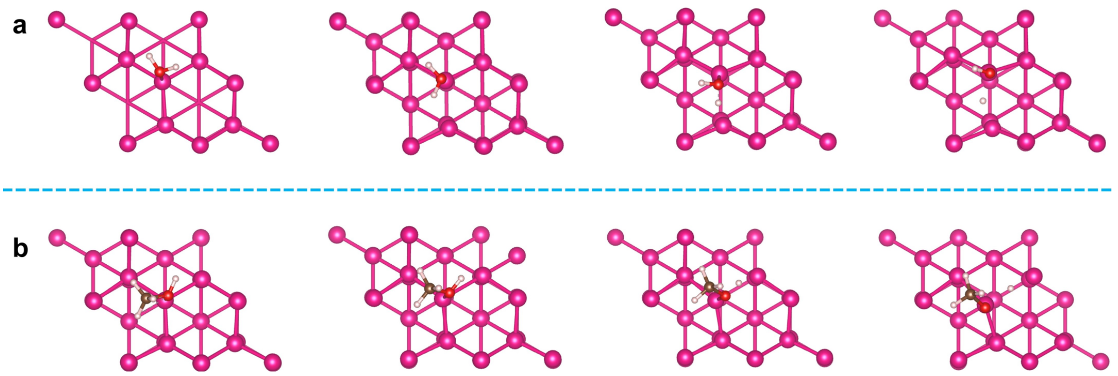

Supplementary Fig. 25 Schematic pathway for the decomposition process of (a) H_2O and (b) CH_3OH molecules on Si (111) facet. Solvent molecules undergo molecule adsorption, molecule rearrangement, molecule breakage, produced H and OR adsorption. Other Zintl phase alloys systems have the similar process to the pure Si.

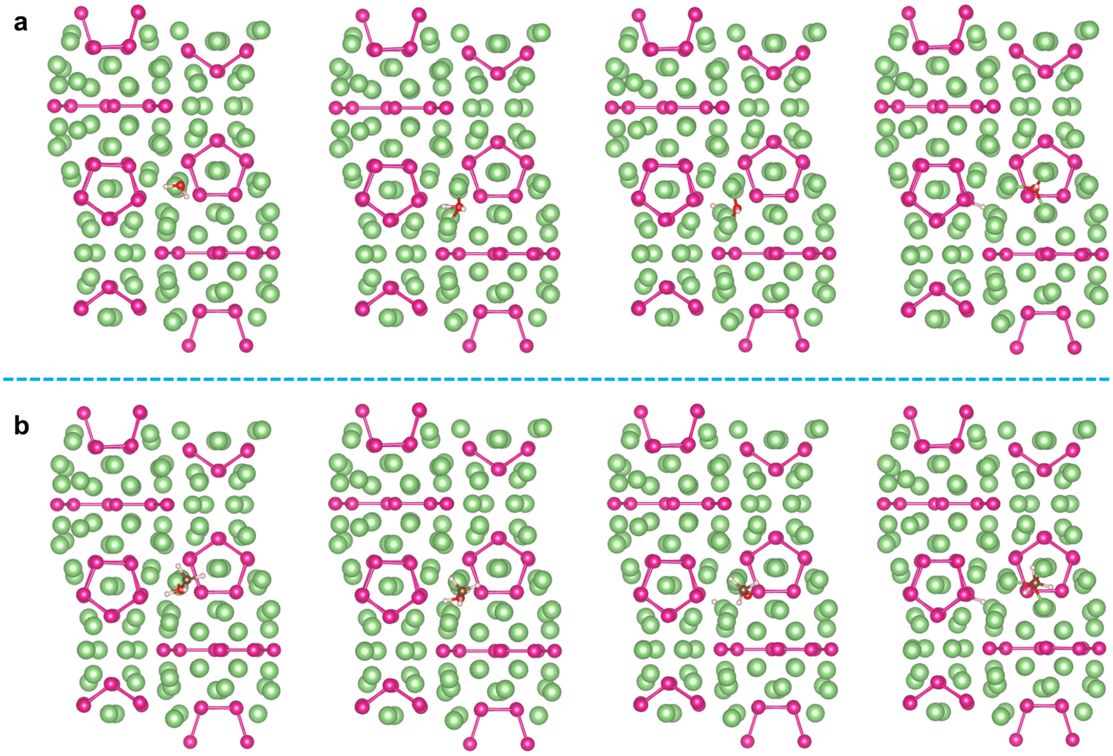

Supplementary Fig. 26 Schematic pathway for the decomposition process of (a) H_2O and (b) CH_3OH molecules on $Li_{12}Si_7$ (100) facet containing Si_5 ring structure.

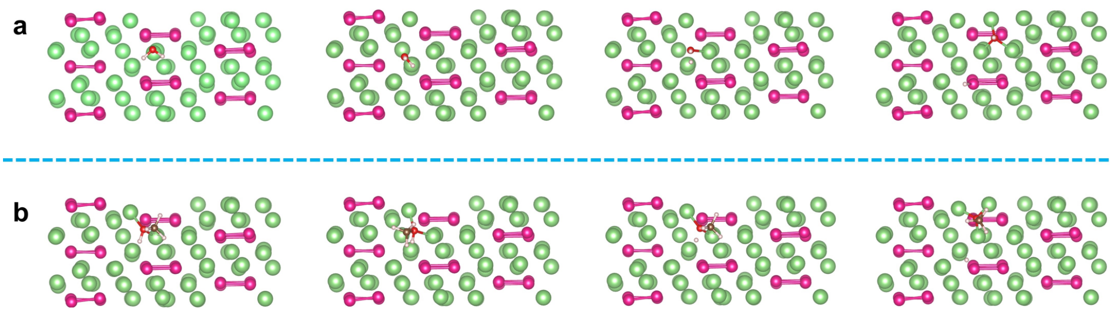

Supplementary Fig. 27 Schematic pathway for the decomposition process of (a) H_2O and (b) CH_3OH molecules on Li_7Si_3 (010) facet containing Si_2 dumbbell structure.

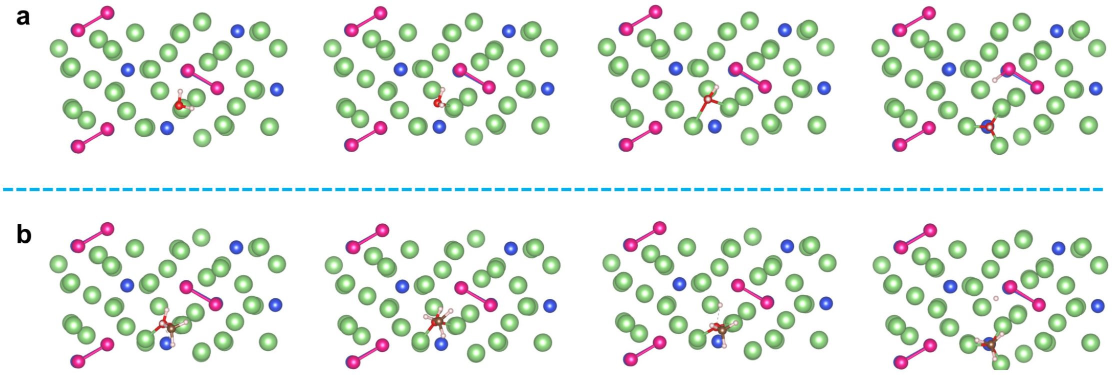

Supplementary Fig. 28 Schematic pathway for the decomposition process of (a) H_2O and (b) CH_3OH molecules on $Li_{13}Si_4$ (100) facet containing Si_2 dumbbell structure (pink atoms).

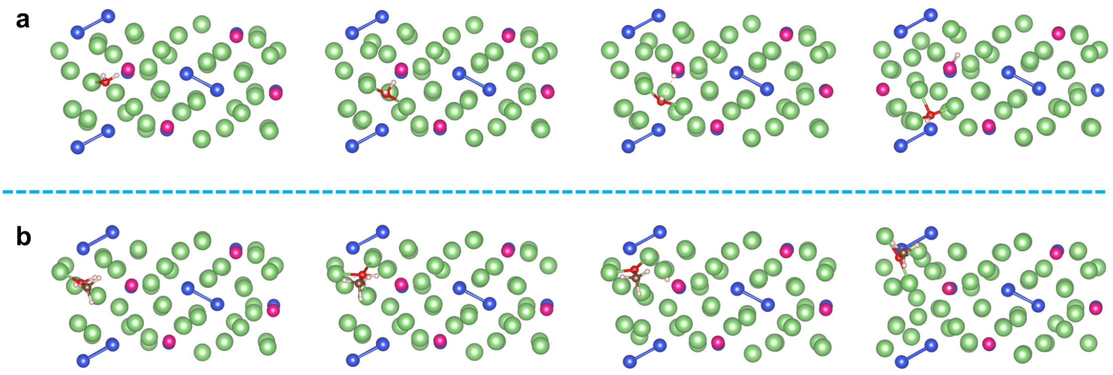

Supplementary Fig. 29 Schematic pathway for the decomposition process of (a) H_2O and (b) CH_3OH molecules on $Li_{13}Si_4$ (100) facet containing Si_1 single atom structure (pink atoms).

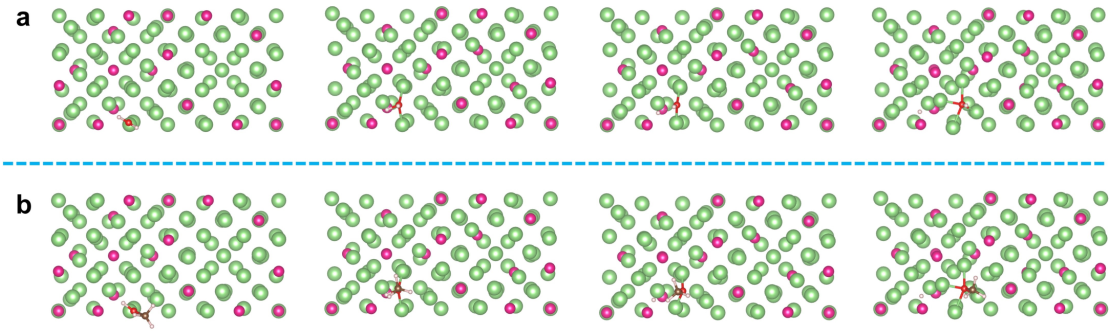

Supplementary Fig. 30 Schematic pathway for the decomposition process of (a) H_2O and (b) CH_3OH molecules on $Li_{21}Si_5$ (110) facet containing Si_1 single atom structure.

a

b

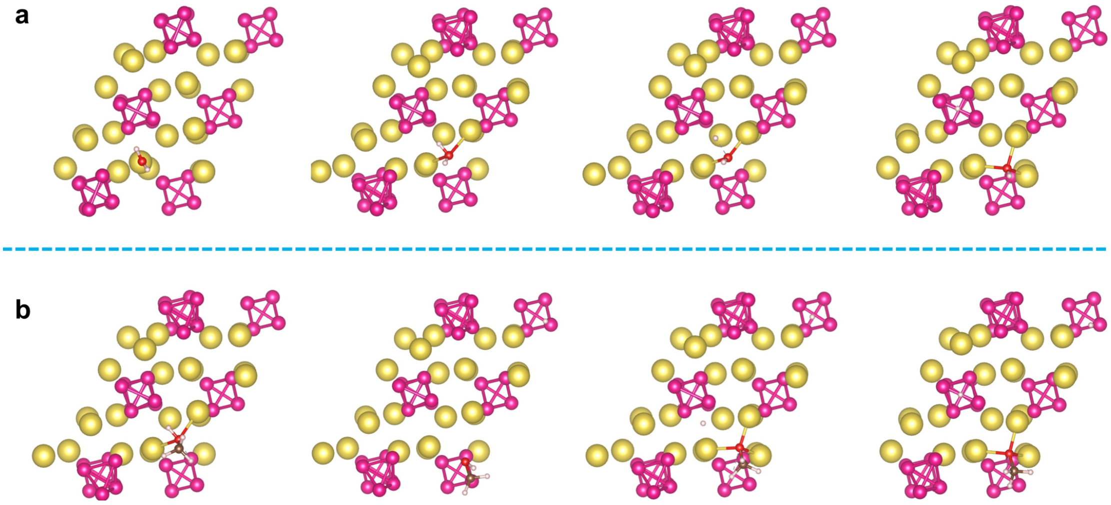

Supplementary Fig. 31 Schematic pathway for the decomposition process of (a) H_2O and (b) CH_3OH molecules on NaSi (010) facet containing Si_4 tetrahedron structure.

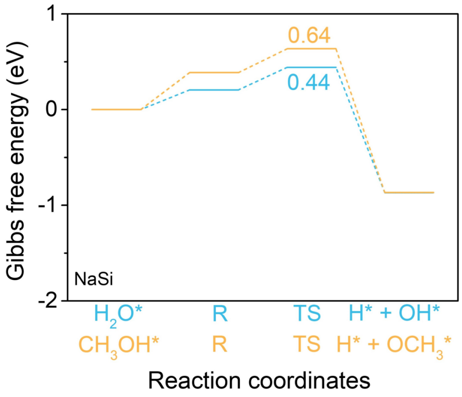

Supplementary Fig. 32 Energy profiles of H_2O and CH_3OH dissociation process on NaSi (010) facet.

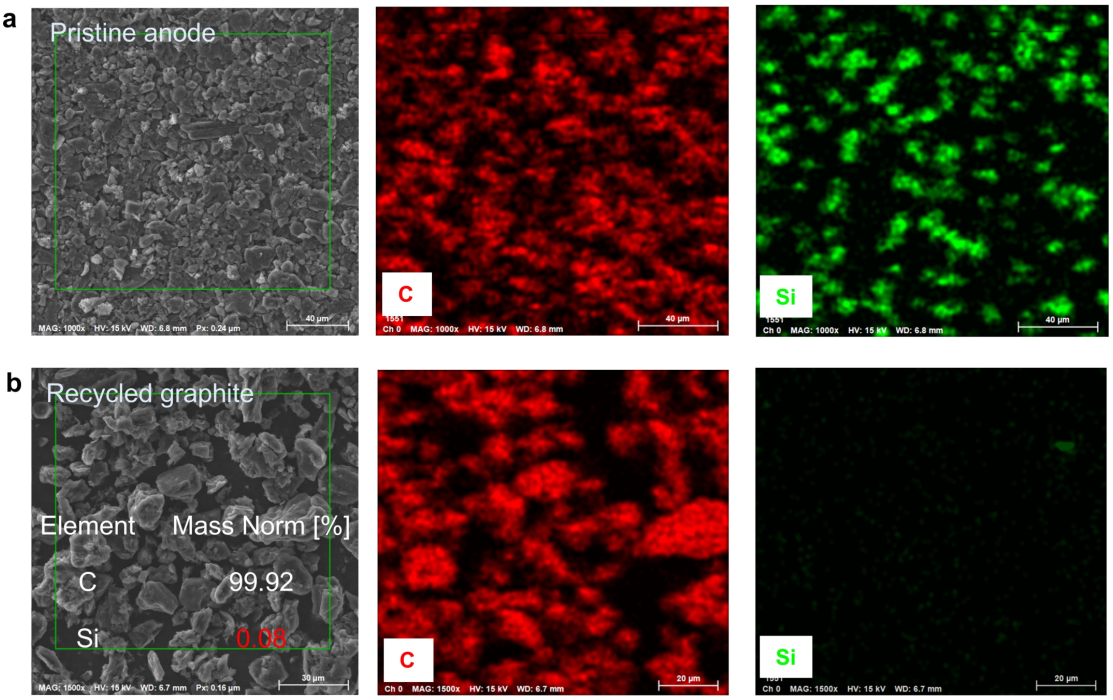

Supplementary Fig. 33 SEM images and EDS mapping results of (a) pristine anode and (b) recycled graphite.

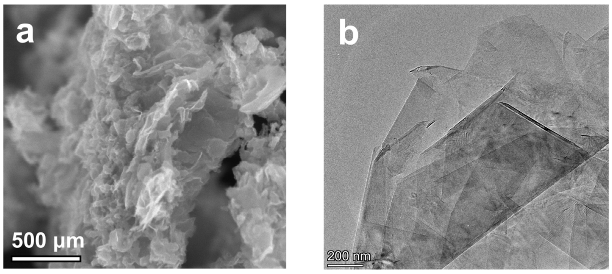

Supplementary Fig. 34 (a) SEM and (b) TEM images of recovered graphene.

Supplementary Table 1 Calculation for mean charge states of Si and Li/Na atoms in pure Si and five Zintl Si-based alloys.

	Si	$Li_{12}Si_7$	Li_7Si_3	$Li_{13}Si_4$	$Li_{21}Si_5$	NaSi
**Si**	0	-1.40	-1.88	-2.62	-3.36	-0.76
**Li**		+0.82	+0.81	+0.81	+0.80	
**Na**	-					+0.76

Supplementary Table 2 Calculation for charge states of various Si structures and Li/Na atoms in pure Si and five Zintl Si-based alloys from the Bader charge analysis.

	Si	$Li_{12}Si_7$	Li_7Si_3	$Li_{13}Si_4$	$Li_{21}Si_5$	NaSi
**Si**	0^(n)^	-1.30 (r)	-1.88 (d)	-2.07 (d)	-3.36 (a)	-0.76 (t)
		-1.65 (s)		-3.16 (a)		
**Li**	-	+0.82	+0.81	+0.81	+0.80	-
**Na**	-					+0.76

Note: (n) network, (r) ring, (s) star, (d) dumbbell, (a) atom, (t) tetrahedron

Supplementary Table 3 Related hydrogen performance information used for the calculation of maximal Si utilization rates.

**Temperature**	**Reaction systems**	**Maximal H_2 Yield (L g^{-1})**	**H_2 yield of Li species (L g^{-1})**	**H_2 yield of Si species (L g^{-1})**	**Theoretical H_2 yield of Si species (L g^{-1})**
25°C	$Li_{21}Si_5$-H_2O	1.643	0.899	0.744	0.856
	$Li_{13}Si_4$-H_2O	1.595	0.786	0.809	0.968
	Li_7Si_3-H_2O	1.539	0.645	0.894	1.107
	$Li_{12}Si_7$-H_2O	1.334	0.525	0.809	1.225
	$Li_{21}Si_5$-CH_3OH	1.739	0.899	0.840	0.856
	$Li_{13}Si_4$-CH_3OH	1.616	0.786	0.830	0.968
	Li_7Si_3- CH_3OH	0.988	0.645	0.343	1.107
	$Li_{12}Si_7$-CH_3OH	0.849	0.525	0.324	1.225
	NaSi-H_2O	0.911	0.224	0.687	1.011
	NaSi-CH_3OH	0.866	0.224	0.642	1.011
10°C	$Li_{21}Si_5$- CH_3OH	1.754	0.899	0.855	0.856
20°C		1.747		0.848	
30°C		1.691		0.792	
40°C		1.656		0.757	
10°C	Li_7Si_3-H_2O	1.566	0.645	0.921	1.107
20°C		1.553		0.908	
30°C		1.521		0.876	
40°C		1.396		0.751	

Supplementary Table 4 Comparison of the hydrogen production performance of various Si-based systems.

Materials	Preparation method	Reaction condition	Hydrogen Generation	Si utilization rate	Ref.
Si nanopowder	ball milling, HF etching and HNO_3 treatment	KOH solution (pH = 12.9), 25°C	0.972 L g^{-1} in 20 min	55.7%	1
Si nanopowder	ball milling and HF etching	pure water, 20°C	< 0.015 L g^{-1} in 400 min	0.9%	2
Si nanocrystal	ball milling and electrochemical HF etching	Water/ethanol (V/V=3:2)	0.820 L g^{-1} in 6000 min	47.0%	3
Layered crystalline silicon	HCl etching of $CaSi_2$	Tap water, 40 °C	0.786 L g^{-1} in 360 min	45.0%	4
Si nanowire	metal-assisted chemical HF etching	pure water, 25°C	0.121 L g^{-1} in 1330 min	6.9%	5
Si/KOH/sucrose composite	ball milling	pure water, 19°C	0.894 L g^{-1} in 180 min	74.7%	6
Si-Li-NaCl	ball milling	pure water	1.213 L g^{-1} in 180 min	73.2%	7
Si nanopowder	ball milling	KOH solution (pH = 9)	0.554 L g^{-1} in 1440 min	31.7%	8
Si nanopowder	ball milling	$NaHCO_3$ + Na_2CO_3 solution (pH = 9), 36°C	0.8 L g^{-1} in 420 min	45.8%	9
H-silicene [a]	7 days' HCl etching of $CaSi_2$ (-25°C) and low temperature sonication	NaOH solution (pH = 12.0)	~1.928 L g^{-1} in ~240 min	100%	10

10 nm Si particles [b]	SiH_4/H_2 laser pyrolysis	8 M KOH solution, room temperature	~2.251 L g^{-1} in 2 min	100%	[11]
$Li_{21}Si_5$ alloy	ball milling	pure water or methanol, 25°C	1.643 L g^{-1} in 1 min/ 1.739 L g^{-1} in 0.6 min	86.9%/98.1 %	**This work**

[a, b] Since the ref.10 and 11 did not provide the specific hydrogen production values of H-silicene and 10 nm Si, their hydrogen yields were estimated based on the research results through the curve recognition software.

Supplementary Table 5 Comparison of the hydrogen evolution performance of Zintl $Li_{21}Si_5$ alloy with the state-of-the-art Si/Mg/Al based materials.

Material	Reaction solution	Reaction temperature	Hydrogen yield	Ref.
$Li_{21}Si_5$	pure water	25°C	1.643 L g^{-1} in 1 min	This work
	methanol	25°C	1.739 L g^{-1} in 0.6 min	
		-10°C	1.613L g^{-1} in 2 min	
		-20°C	1.366 L g^{-1} in 2 min	
		-30°C	1.254 L g^{-1} in 2 min	
		-40°C	1.091 L g^{-1} in 26 min	
Si-CaH_2	pure water	25°C	1.216 L g^{-1} in 10 min	c
Si/KOH/sucrose	pure water	19°C	0.894 L g^{-1} in 180 min	6
Si-Li-NaCl	pure water	30°C	1.213 L g^{-1} in 180 min	7
Li–$CaSi_2$	0.5 M NaF	25°C	0.590 L g^{-1} in 20 min	13
Mg-Mg_2Si	0.5 M $MgCl_2$	20°C	0.788 L g^{-1} in 40 min	14
Si-$NaBH_4$-$AlCl_3$	pure water	25°C	0.818 L g^{-1} in 90 min	15
Nano Si	water/ethanol	-40°C	0.343 L g^{-1} in 150 min	3
MCR Mg-GO	3.5wt% NaCl	25°C	0.934 L g^{-1} in 10 min	16
Mg-Ni_3Fe/RGO	3.5wt% NaCl	30°C	0.812 L g^{-1} in 1 min	17
Mg–Mo_2C	sea water	25°C	0.851 L g^{-1} in 10 min	18
Mg-Mg_2NiH_4	3.5wt% NaCl	30°C	0.845 L g^{-1} in 0.3 min	19
Mg-MoO_3	3.5wt% NaCl	25°C	0.842 L g^{-1} in 10 min	20

$CaMg_2$	methanol	-20°C	0.609 L g^{-1} in 100 min	21
La-doped Mg-Ni	3.5wt% NaCl	18°C	0.824 L g^{-1} in 20 min	22
Mg30La-EG	3.5wt% NaCl	25°C	0.649 L g^{-1} in 90 min	23
Al/GIS/NaCl/(CS+CN)	23wt%% snow-melting agent aqueous	-20°C	1.095 L g^{-1} in 60 min	24
Al alloy/NaCl/g-C_3N_4	23wt% NaCl	-20°C	1.006 L g^{-1} in 6 min	25
Al/Ni0.1/Cu0.1	pure water	25°C	0.928 L g^{-1} in 10 min	26
Al-Ga-In-Sn	tap water	25°C	1.146 L g^{-1} in 10 min	27
Al-NaH	pure water	31°C	0.800 L g^{-1} in 10 min	28
Al–Bi	pure water	35°C	1.120 L g^{-1} in 10 min	29

Supplementary Table 6 The theoretical exothermicity of different Li-Si alloys in pure water and methanol.

Reaction equation	△H (kJ mol^{-1})	Heating (kJ g_{alloy}^{-1})
$Li_{12}Si_7 + 21.24H_2O \rightarrow 12LiOH + 4.62SiO_2 +2.38Si+15.24H_2$	-3190.7	11.4
$Li_7Si_3 + 11.848H_2O \rightarrow 7LiOH + 2.424SiO_2 + 0.576Si+8.348H_2$	-1777.5	13.4
$Li_{13}Si_4 + 19.688H_2O \rightarrow 13LiOH + 3.344SiO_2 + 0.656Si + 13.188H_2$	-2986.4	14.7
$Li_{21}Si_5 + 29.69H_2O \rightarrow 21LiOH + 4.345SiO_2 + 0.655Si + 19.19H_2$	-4600.0	16.1
$Li_{12}Si_7 + 19.392CH_3OH \rightarrow 12LiOCH_3 + 1.848Si(OCH_3)_4 + 5.152Si+9.696H_2$	-2373.4	8.5
$Li_7Si_3 + 10.72CH_3OH \rightarrow 7LiOCH_3 + 0.93Si(OCH_3)_4 + 2.07Si+5.36H_2$	-1349.5	10.2
$Li_{13}Si_4 + 26.712CH_3OH \rightarrow 13LiOCH_3 + 3.428Si(OCH_3)_4 + 0.572Si + 13.356H_2$	-3020.7	14.9
$Li_{21}Si_5 + 40.62CH_3OH \rightarrow 21LiOCH_3 + 4.905Si(OCH_3)_4 + 0.095Si+20.31H_2$	-4830.8	16.9

[a] $Li_{21}Si_5$, △H: -555.2 kJ mol^{-1} | Factstage software for |;
[b] $Li_{13}Si_4$, △H: -417.7 kJ mol^{-1} | Factstage software for |;
[c] Li_7Si_3, △H: -258.2 kJ mol^{-1} | Factstage software for |;
[d] $Li_{12}Si_7$, △H: -450.1 kJ mol^{-1} | Factstage software for |;
[e] H_2O, △H: -285.8 kJ mol^{-1} | Factstage software for |;
[f] CH_3OH, △H: -238.7 kJ mol^{-1} | Factstage software for |;
[h] LiOH, △H: -464 kJ mol^{-1} | Factstage software for |;
[i] $LiOCH_3$, △H: -433 kJ mol^{-1} | Ref [30]. for |;
[j] SiO_2, △H: -896.8 kJ mol^{-1} | Factstage software for |;
[k] $Si(OCH_3)_4$, △H: -1221 kJ mol^{-1} | Ref [31]. for |;
[l] Si, △H: 0 kJ mol^{-1};
[m] H_2, △H: 0 kJ mol^{-1};

Supplementary References

1 Imamura, K., Kimura, K., Fujie, S. & Kobayashi, H. Hydrogen generation from water using Si nanopowder fabricated from swarf. J. Nanopart. Res. **18**, 116, doi:10.1007/s11051-016-3418-x (2016).

2 Kobayashi, Y., Matsuda, S., Imamura, K. & Kobayashi, H. Hydrogen generation by reaction of Si nanopowder with neutral water. J Nanopart Res **19**, 176, doi:10.1007/s11051-017-3873-z (2017).

3 Mussabek, G. et al. Kinetics of Hydrogen Generation from Oxidation of Hydrogenated Silicon Nanocrystals in Aqueous Solutions. Nanomaterials **10**, 1413, doi:10.3390/nano10071413 (2020).

4 Ma, W. et al. Robust hydrogen generation over layered crystalline silicon materials via integrated H_2 evolution routes. Int. J. Hydrogen Energy **45**, 19007-19016, doi:10.1016/j.ijhydene.2020.05.095 (2020).

5 Ning, R. et al. On-demand production of hydrogen by reacting porous silicon nanowires with water. Nano Res. **13**, 1459-1464, doi:10.1007/s12274-020-2734-8 (2020).

6 Xu, L. et al. Ball-milled Si powder for the production of H_2 from water for fuel cell applications. Int. J. Hydrogen Energy **41**, 12730-12737, doi:https://doi.org/10.1016/j.ijhydene.2016.05.181 (2016).

7 Liu, M. et al. In Situ Formation of Li_2SiO_3-Li-NaCl Interface on Si and Its Effect on Hydrogen Evolution. ACS Appl. Mater. Interfaces **15**, 20917-20924, doi:10.1021/acsami.2c23285 (2023).

8 Kobayashi, Y., Fujie, S., Imamura, K. & Kobayashi, H. Structure and hydrogen generation mechanism of Si-based agent. Appl. Surf. Sci. **536**, 147398, doi:10.1016/j.apsusc.2020.147398 (2021).

9 Kobayashi, Y. et al. Renoprotective and neuroprotective effects of enteric hydrogen generation from Si-based agent. Sci Rep **10**, 5859, doi:10.1038/s41598-020-62755-9 (2020).

10 You, Y. et al. Water-Enabled H_2 Generation from Hydrogenated Silicon Nanosheets for Efficient Anti-Inflammation. J Am Chem Soc **144**, 14195-14206, doi:10.1021/jacs.2c04412 (2022).

11 Erogbogbo, F. et al. On-Demand Hydrogen Generation using Nanosilicon: Splitting Water without Light, Heat, or Electricity. Nano Lett. **13**, 451-456, doi:10.1021/nl304680w (2013).

12 Liu, M. et al. Local high concentration alkali field accelerated silicon hydrolysis for hydrogen production. Chem. Eng. J. **475**, 146201, doi:https://doi.org/10.1016/j.cej.2023.146201 (2023).

13 Xie, J. et al. A comprehensive study on hydrolysis and electrochemistry of Li–Ca–Si alloys. J. Power Sources **614**, 234952, doi:10.1016/j.jpowsour.2024.234952 (2024).

14 Tan, Z. et al. Hydrogen generation by hydrolysis of Mg-Mg_2Si composite and enhanced kinetics performance from introducing of $MgCl_2$ and Si. Int. J. Hydrogen Energy **43**, 2903-2912, doi:10.1016/j.ijhydene.2017.12.163 (2018).

15 Feng, Z. et al. Promoted hydrolysis performances and mechanism of Si-$NaBH_4$-$AlCl_3$ in deionized water. Chin. J. Chem. Eng. **28**, 3136-3144, doi:10.1016/j.cjche.2020.08.019 (2020).

16 Bai, J. et al. Hydrolytic hydrogen production behavior in NaCl solution of Mg and Mg-GO composite synthesized by mechanochemical reaction. J. Alloys Compd. **969**, 172473, doi:10.1016/j.jallcom.2023.172473 (2023).

17 Liu, J. et al. Supra Hydrolytic Catalysis of Ni_3Fe/rGO for Hydrogen Generation. Adv. Sci. **9**, e2201428, doi:10.1002/advs.202201428 (2022).

18 Naseem, K. et al. A reusable dual functional Mo_2C catalyst for rapid hydrogen evolution by Mg hydrolysis. J. Mater. Chem. A, 19328–19337, doi:10.1039/D3TA02163G (2023).

19 Zhou, C. et al. Enhanced hydrogen generation via hydrolysis of Mg–Mg_2NiH_4 system. J. Power Sources **476**, 228499, doi:10.1016/j.jpowsour.2020.228499 (2020).

20 Huang, M. et al. Hydrogen generation via hydrolysis of magnesium with seawater using Mo, MoO_2, MoO_3 and MoS_2 as catalysts. J. Mater. Chem. A **5**, 8566-8575, doi:10.1039/c7ta02457f (2017).

21 Ma, M. et al. Kinetically Controllable Hydrogen Generation at Low Temperatures by the Alcoholysis of $CaMg_2$ -Based Materials in Tailored Solutions. ChemSusChem **13**, 2709-2718, doi:10.1002/cssc.202000089 (2020).

22 Hou, X. et al. H_2 generation kinetics/thermodynamics and hydrolysis mechanism of high-performance La-doped Mg-Ni alloys in NaCl solution—A large-scale and quick strategy to get hydrogen. J. Magnesium Alloys **9**, 1068-1083, doi:10.1016/j.jma.2020.05.020 (2020).

23 Hou, X. et al. Investigation on environmental stability and hydrolytic hydrogen production behavior of Mg30La alloy modified by activated carbon/expandable graphite. Int. J. Hydrogen Energy **49**, 1145-1160, doi:10.1016/j.ijhydene.2023.06.243 (2024).

24 Xu, H. et al. High efficiency Al-based multicomponent composites for low-temperature hydrogen production and its hydrolysis mechanism. Int. J. Hydrogen Energy **48**, 26260-26275, doi:10.1016/j.ijhydene.2023.03.338 (2023).

25 Su, M. et al. Thermodynamics, kinetics and reaction mechanism of hydrogen production from a novel Al alloy/NaCl/g-C_3N_4 composite by low temperature hydrolysis. Energy **218**, 119489, doi:10.1016/j.energy.2020.119489 (2021).

26 Guo, J. et al. Enhanced hydrogen generation from Al-water reaction mediated by metal salts. Int. J. Hydrogen Energy **46**, 3453-3463, doi:10.1016/j.ijhydene.2020.10.220 (2021).

27 Guan, X., Zhou, Z., Luo, P., Wu, F. & Dong, S. Effects of preparation method on the hydrolytic hydrogen production performance of Al-rich alloys. J. Alloys Compd. **796**, 210-220, doi:10.1016/j.jallcom.2019.05.053 (2019).

28 Hammad, A. et al. Aluminum hydrolysis for hydrogen generation enhanced by sodium hydride. Int. J. Hydrogen Energy **77**, 138-148, doi:10.1016/j.ijhydene.2024.06.174 (2024).

29 Liu, Z. et al. Study on the hydrogen generation performance and hydrolyzates of active aluminum composites. Int. J. Hydrogen Energy **47**, 1701-1709, doi:10.1016/j.ijhydene.2021.10.113 (2022).

30 Leal, J. P. & Simões, J. A. M. Standard molar enthalpies of formation of lithium alkoxides. J. Organomet. Chem. **460**, 131-138, doi:https://doi.org/10.1016/0022-328X(93)83138-L (1993).

31 Voronkov, M. G. et al. Thermochemistry of organosilicon compounds: I. Triorganyl-, tetraorganyl-, organylorganoxy- and tetraorganoxy-silanes. J. Organomet. Chem. **345**, 27-38, doi:https://doi.org/10.1016/0022-328X(88)80231-6 (1988).
