# Supplementary material for: Activating silicon for high hydrogen conversion and sustainable anode recovery
Source: Nat Commun. 2025 Aug 20;16:7772. doi: 10.1038/s41467-025-63086-x (PMC12368011; doi:10.1038/s41467-025-63086-x)
Supplement: Supplementary file 2 — Description of Additional Supplementary Information [file 41467_2025_63086_MOESM2_ESM.pdf]

Description of Additional Supplementary Files

File Name: Supplementary Movie 1
Description: Hydrolysis of charged electrode.
